# Exosomes-Mediated Signaling Pathway: A New Direction for Treatment of Organ Ischemia-Reperfusion Injury

**DOI:** 10.3390/biomedicines12020353

**Published:** 2024-02-02

**Authors:** Yanying Wang, Ruojiao Xu, Yujia Yan, Binyu He, Chaoyi Miao, Yifeng Fang, Haitong Wan, Guoying Zhou

**Affiliations:** 1 The Second Clinical Medical College, Zhejiang Chinese Medical University, 548 Binwen Road, Hangzhou 310053, China; 15605809861@163.com (Y.W.); 15757996320@163.com (B.H.); 19558153512@163.com (C.M.); 2 College of Life Science, Zhejiang Chinese Medical University, 548 Binwen Road, Hangzhou 310053, China; 18268922805@163.com (R.X.); yanyujiay@163.com (Y.Y.); fangyifeng2022@163.com (Y.F.)

**Keywords:** ischemia-reperfusion, exosomes, signaling pathway, reperfusion injury

## Abstract

Ischemia reperfusion (I/R) is a common pathological process which occurs mostly in organs like the heart, brain, kidney, and lung. The injury caused by I/R gradually becomes one of the main causes of fatal diseases, which is an urgent clinical problem to be solved. Although great progress has been made in therapeutic methods, including surgical, drug, gene therapy, and transplant therapy for I/R injury, the development of effective methods to cure the injury remains a worldwide challenge. In recent years, exosomes have attracted much attention for their important roles in immune response, antigen presentation, cell migration, cell differentiation, and tumor invasion. Meanwhile, exosomes have been shown to have great potential in the treatment of I/R injury in organs. The study of the exosome-mediated signaling pathway can not only help to reveal the mechanism behind exosomes promoting reperfusion injury recovery, but also provide a theoretical basis for the clinical application of exosomes. Here, we review the research progress in utilizing various exosomes from different cell types to promote the healing of I/R injury, focusing on the classical signaling pathways such as PI3K/Akt, NF-κB, Nrf2, PTEN, Wnt, MAPK, toll-like receptor, and AMPK. The results suggest that exosomes regulate these signaling pathways to reduce oxidative stress, regulate immune responses, decrease the expression of inflammatory cytokines, and promote tissue repair, making exosomes a competitive emerging vector for treating I/R damage in organs.

## 1. Introduction

### 1.1. Overview of Ischemia-Reperfusion Injury

**Figure 1:**
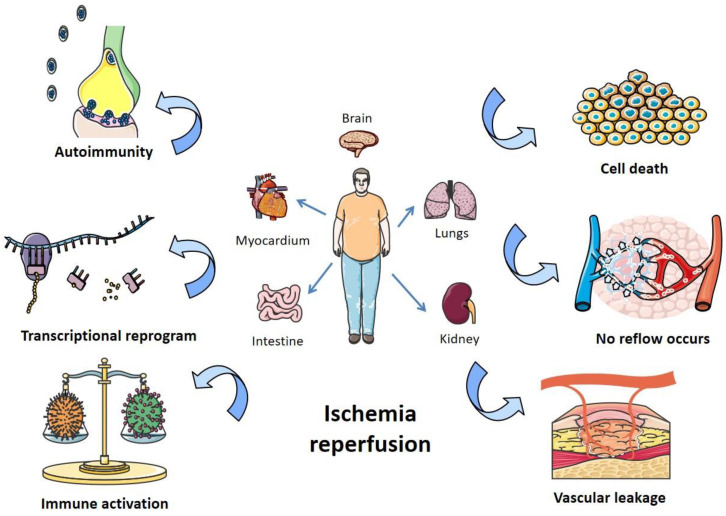
Pathogenesis of organ I/R injury. I/R damages organs like the heart, lungs, kidneys, and intestines, mainly by affecting autoimmunity, transcriptional recoding, immune activation, apoptosis, blocking blood return, and vascular leakage.

Ischemia-reperfusion (I/R) injury is a pathological process that can occur in many organs of the human body, accompanied by severe cell damage and inflammatory reactions [1]. In recent years, it has become one of the most common causes of disability and death worldwide [2]. There is a lot of evidence that tissue damage is caused not by ischemia itself, but by the sudden resumption of blood supply after a period of ischemia [3]. Organ ischemia-reperfusion injury often occurs in traumatic shock, surgery, organ transplantation, burns, and other blood circulation disorders [4]. The main organs involved in vascular reperfusion injury are the heart, brain, liver, and kidney, and it can also induce systemic inflammation and eventually lead to multiple organ failure [5]. However, not all organs with ischemia will have I/R injury after blood flow recovery, and many factors can affect its occurrence and severity. For example, if the ischemia time is short, or collateral circulation is easy to form after ischemia, there may be no significant reperfusion injury after the restoration of blood supply. Because oxygen readily accepts electrons and forms oxygen free radicals, organs with a high oxygen demand are more prone to reperfusion injury [6]. In addition, appropriately reducing the temperature and pH value of the perfusion solution and reducing the content of Ca^2+^ and Na^+^ in the perfusion solution are conducive to alleviating the reperfusion injury [7]. The pathogenesis of organ I/R injury is summarized in Figure 1.

### 1.2. Occurrence and Mechanism of Ischemia-Reperfusion Injury

**Figure 2:**
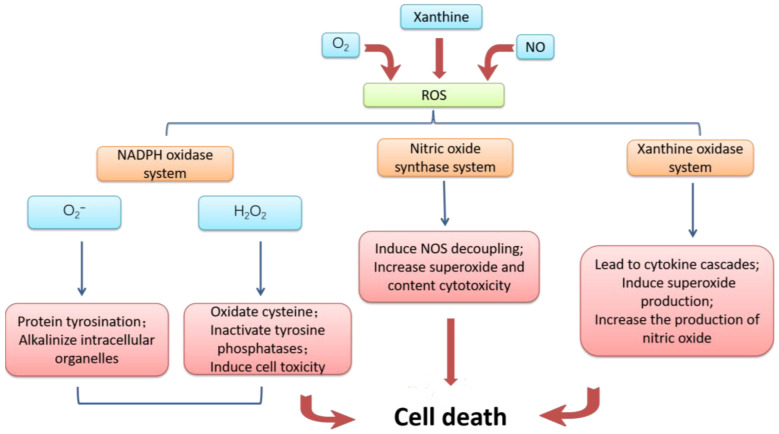
Mechanism of cell damage induced by reactive oxygen species. Excessive accumulation of reactive oxygen caused by I/R and conversion to NADPH oxidase, nitric oxide synthase inhibitor, and xanthine oxidase resulting in cell apoptosis.

The mechanism of ischemia-reperfusion injury is complex, and the following four aspects are mainly responsible according to the current studies. First of all, the damage to microvessels and parenchymal organs caused by ischemic tissue reperfusion is mainly caused by oxidative stress [8]. Ischemia leads to an inadequate supply of oxygen and nutrients, and cells are unable to produce enough energy. When reperfusion occurs, the sudden supply of oxygen and nutrients increases the degree of oxidative stress within the cell, resulting in the production of excessive reactive oxygen species and other oxidizing substances [9]. The mechanism of cell apoptosis accelerated by massive reactive oxygen is summarized in Figure 2. The synthesis ability of antioxidant enzymes that can scavenge free radicals in ischemic tissue is greatly weakened, and the strong oxidation effect directly damages vascular endothelial cells and tissue cells, thus exacerbating the damage of free radicals to the tissue after I/R [10].

**Figure 3:**
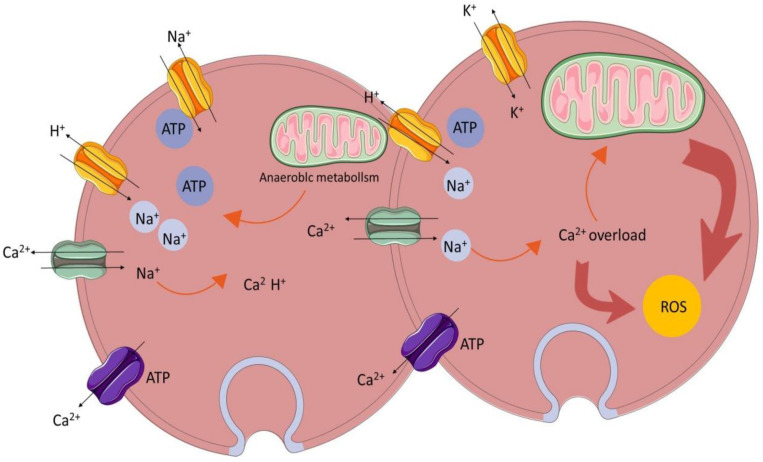
Calcium overload.

The homeostasis and regulation of the calcium ion concentration in and outside cells are important conditions for maintaining cell function [11]. Calcium overload is the phenomenon of an abnormal intracellular calcium ion concentration leading to structural damage and dysfunction in the functional metabolism [12]. The mechanism of calcium overload is summarized in Figure 3. When I/R injury occurs, the local intracellular calcium ion concentration increases significantly, resulting in the disturbance of calcium ion homeostasis and an insufficient mitochondrial oxidation capacity [13], thus activating phospholipase to damage other organelles or activating calcium-dependent protease activity, as well as increasing the production of oxygen free radicals [14].

In addition, inflammatory responses and immune cell activation also play important roles in the occurrence of ischemia-reperfusion injury [15]. During reperfusion, under the influence of excessive oxidizing substances, immune cells release a large number of inflammatory mediators, causing cell damage in the surrounding tissue [16]. Meanwhile, a large number of free radicals and lysosomal enzymes released by white blood cells make the vascular endothelial cells swell, the microvascular diameter narrow, and permeability increase, meaning that reperfusion blood flow cannot be re-routed [17]. In addition, I/R injury can result in mitochondrial dysfunction, including a reduced mitochondrial permeability, DNA damage, and impaired respiratory chain function. These pathological changes lead to reduced intracellular ATP production, limited mitochondrial respiratory function, and increased oxidative stress [18]. As a result, if I/R injury is not treated properly, it can progress to irreversible loss of organ function and eventually threaten life.

### 1.3. Current Therapeutic Strategies for Ischemia-Reperfusion Injury

Therefore, reducing inflammation, scavenging free radicals, maintaining calcium homeostasis, and antioxidant therapy are the keys to reducing ischemia-reperfusion injury. In recent years, more and more medical interventions and strategies have been actively applied to minimize I/R injury. However, due to the complex pathologic factors of I/R injury, traditional treatment strategies such as blood pressure control, detumescence, and dehydration are of limited effectiveness [19,20]. Recently, advanced therapies such as ischemic preconditioning, ischemic post-treatment, antioxidant, hypothermia, and stem cell therapy have emerged as potential treatments for I/R injury [21]. Ischemic preconditioning (IPC) is considered as an effective treatment strategy for central nervous system diseases. It causes transient ischemia and the reperfusion of organs or tissues through drugs, intermittent blood flow blockade, and other methods, with the aim of inducing adaptive changes in cells and improving tolerance to subsequent, more severe I/R events [22]. Similar to preconditioning, ischemic posttreatment aims to reduce the harmful effects of reperfusion by activating protective signaling pathways and reducing apoptosis [23]. Several drugs and compounds have shown promise in reducing I/R injury. These include antioxidants (vitamin C), anti-inflammatory agents (corticosteroids), and mitochondrial protectors (CoQ10), which are used to reduce oxidative stress and maintain mitochondrial function [24]. Cooling damaged tissues or organs can slow metabolic processes and reduce oxygen and energy consumption, thus reducing tissue damage. Hypothermia therapy has been shown to be beneficial in reducing I/R injury, especially in the brain and heart [25].

Cell therapy is a new approach to the treatment of ischemia-reperfusion injury, and its effects are constantly being studied and explored. Stem cells can promote the healing of I/R injury by repairing tissue cell damage and stimulating the body’s own cell regeneration, secreting vascular growth factors to improve blood supply and releasing apoptosis inhibitors to reduce the occurrence of apoptosis [26]. However, its short survival time and low antigen expression in the body can not be underestimated. In recent years, researchers have found that exosomes from different stem cells can not only overcome the limitations of stem cells, but also retain the therapeutic effect of stem cells [27]. Therefore, they have become one of the research focuses in the treatment of I/R injury.

### 1.4. Exosomes Mediate Organ Ischemia-Reperfusion Injury through Different Signaling Pathways

Exosomes are vesicles between 40 nm and 160 nm in diameter that are released into the blood from cells. Exosomes released into cells can participate in immune response, cell migration, cell differentiation, antigen presentation, and other processes [28]. Exosomes play an important role in cell communication, transporting proteins, mRNA, lipids, and other exogenous substances to recipient cells and even crossing the blood–brain barrier (BBB) [29]. On the other hand, exosomes play a compelling role in immune response. Exosomes can induce different signaling pathways through microRNAs (miRNAs) carried by them or ligands existing on the surface to play a role in promoting tissue repair and regeneration, alleviating inflammation, and alleviating organ damage [30]. More and more evidence shows that the bioactive substances carried by exosomes reflect the state of the disease, making them a marker for the diagnosis of hypoxic diseases [31]. For example, miRNA-328-3p carried by serum exosomes in brain I/R injury plays a key predictive role in the short-term prognosis of stroke [32]. Hu et al. showed that, in the presence of reactive oxygen species, the release of exosomes derived from mesenchymal stem cells can enhance vascular healing and promote the formation and proliferation of endothelial cells in rats with renal ischemia-reperfusion injury [33]. Furthermore, exosome bioengineering has attracted more and more attention in purification, biological characterization, and pharmacological applications, and it can be considered that exosomes have great potential in the diagnosis and treatment of I/R injury [34]. Different cell-derived exosomes, whose functions are affected by various physical, chemical, and biological processes, also play different roles in the repair of ischemia-reperfusion injury [35].

The signaling pathway refers to a series of enzymatic reaction pathways that can transmit extracellular molecular signals through the cell membrane to exert effects in cells. Hormones, neurotransmitters, and other extracellular biological signals are perceived by their receptors and transformed into intracellular signals, which are then amplified and regulated by signal cascades, and finally produce a series of comprehensive cellular responses [36]. Once released, exosomes send signals to other cells to mediate intercellular communication in three ways: First, exosome membrane proteins can bind to receptors on their target cell membranes, activating signaling pathways in their target cells. Second, exosome membrane proteins are cleaved by proteases in the extracellular matrix and then bind to receptors on the target cell membrane, thereby activating intracellular signaling pathways. Third, exosomes can directly fuse with target cell membranes to regulate cell signaling through the non-selective release of proteins and miRNAs [37]. There is increasing evidence that exosomes regulate ischemia-reperfusion injury healing in organs through different signaling pathways.

Therefore, this paper aims to provide an update on the use of exosomes in the treatment of ischemia-reperfusion injury in organs based on various signaling pathways [38], and to elucidate the importance of these pathways in the mechanism of reperfusion injury healing and how exosomes are involved in these processes.

## 2. The Signaling Pathway of Exosomes in Promoting Organ Ischemia-Reperfusion Injury Recovery

The signaling pathways mediated by exosomes that are conducive to the healing of ischemia reperfusion injury mainly include PI3K/Akt, NF-κB, Nrf2, PTEN, Wnt, MAPK, toll-like receptor, and AMPK, etc. Examples of exosomes from different sources and their roles, mechanisms, and signaling pathways associated with ischemia-reperfusion injury repair are presented in Table 1.

### 2.1. PI3K/Akt Signaling Pathway

The PI3K/Akt pathway consists of phosphatidylinositol-3-kinase (PI3K) and the serine/threonine kinase Akt (also known as protein kinase B or PKB), activated by receptor tyrosine kinase and cytokine receptors [83]. The pathway can reduce the degree of cell damage and promote cell survival by reducing the expression of inflammatory factors [84]. In addition, PI3K/Akt can activate nitric oxide synthase to push the production and removal of oxygen free radicals in the body into a dynamic equilibrium state [85].

Recent studies have shown that some factors involved in the PI3K/Akt signaling pathway also contribute to the treatment of ischemia-reperfusion injury. It can reduce apoptosis by regulating apoptosis-related proteins and inhibiting the activation of apoptosis pathways. In addition, Akt activation can inhibit inflammatory responses and oxidative stress and reduce cell damage [86]. Chen et al. showed that Danhong Injection can protect the nerves of rats with I/R injury by activating the PI3K/Akt signaling pathway [87]. Moreover, methyleugenol mediates the PI3K/Akt pathway, which acts on injured hepatocytes in vitro and in vivo to promote cell survival and relieve LiRI-induced liver injury and inflammation [88]. Exosomes from different types of cells play an important role in the treatment of ischemia-reperfusion injury in different organs through the PI3K/Akt regulatory pathway.

#### 2.1.1. Brain

Exosomes play an important role in protecting the nervous system from ischemia-reperfusion injury. In the study by Zhang et al., it was shown that stem-cell-derived exosomes can reduce the expression of inflammatory cytokines and inhibit neuronal apoptosis in focal cerebral ischemia-reperfusion models by activating the PI3K/Akt signaling pathway, thus ameliorating brain injury caused by cerebral ischemia-reperfusion [39].

#### 2.1.2. Heart

The PI3K/Akt pathway mediated by exosomes has a good therapeutic effect on myocardial ischemia-reperfusion injury. In addition, researchers have declared that the highly expressed exosome miRNA-497 isolated from human bone marrow mesenchymal stem cells (HBMSCs) attenuates myocardial ischemia-reperfusion injury by reducing HLA complex P5 (HCP5) expression in cardiomyocytes’ PI3K/Akt pathway [40]. Similarly, exosomes derived from HBMSCs with a high expression of miRNA-486-5p promote the proliferation of H9C2 cells by regulating the PI3K/Akt signaling pathway in vitro, inhibiting the apoptosis of injured myocardial cells in a rat myocardial ischemia-reperfusion injury (MIRI) model under hypoxia/reoxygenation (H/R) conditions [41]. It provides a new therapeutic target for protecting myocardial injury. Moreover, plasma exosomes (P-exos) treated with ischemic preconditioning have improved cardiac function by activating the PI3K/Akt pathway to up-regulate the expression of Bcl-2 (B-cell lymphoma-2) and reduce the expression of inflammatory cytokines [42].

#### 2.1.3. Liver

Studies have confirmed that the PI3K/Akt pathway has a positive effect in the treatment of hepatic ischemia-reperfusion injury. The paper by Zheng et al. proved exosomes produced by bone-marrow-derived dendritic cells (BMDCs) can enhance anti-inflammatory cytokine secretion and regulate the balance between different types of T cells by activating the PI3K/Akt pathway, representing a novel strategy for the treatment of liver ischemia-reperfusion injury [43].

#### 2.1.4. Genital System

Exosomes are of great significance to the reproductive system through the PI3K/AKT pathway. In studies by Liu et al., adipose-derived stromal cells (ADSCs) induced spermatocyte proliferation and migration by releasing exosomes, reducing malondialdehyde (MDA) levels and increasing superoxide dismutase (SOD) levels. Meanwhile, exosomes reduce the expression of oxidative stress and inflammatory factors through PI3K/Akt signaling, thereby improving testicular torsion injury [44].

The extracellular vesicles derived from human umbilical cord mesenchymal cells (HUCMCs) inhibit the activation of caspase-3 and endoplasmic reticulum (ER) stress markers expression by regulating the PI3K/Akt pathway in H/R models cultured by H9C2. Thus, it can reduce cell apoptosis and play a role in improving ischemia-reperfusion injury both in vivo and in vitro [45].

However, excessive activation of the PI3K/Akt signaling pathway may also have a negative impact on I/R injury. Excessive Akt activation may lead to mitochondrial dysfunction and calcium ion homeostasis imbalance, promote inflammatory response and cytokine release, and lead to increased tissue damage and cell death [85]. Therefore, balancing and regulating the activity of the PI3K/Akt signaling pathway is important for the treatment of I/R injury.

### 2.2. NF-κB Signaling Pathway

Nuclear factor kappa-B (NF-κB) protein is an important nuclear transcription factor, which plays an important role in physiological and pathological processes such as inflammation and immunity [89]. In ischemia-reperfusion injury, the role of different signaling pathways may vary depending on specific conditions and tissue types. However, the NF-κB signaling pathway is generally considered to be one of the most important.

As a typical pro-inflammatory signaling pathway, NF-κB is involved in the release of pro-inflammatory cytokines, chemokines, and adhesion molecules [90], and its activation has been widely observed in the pathological processes associated with I/R injury. For instance, physcion protects neuronal cells from I/R damage by reducing oxidative stress and inflammation levels in vitro and in vivo by inhibiting the NF-kB pathway [91]. However, too mild or too severe of an inflammatory response is not conducive to injury recovery [92]. Therefore, binding exosomes to regulate the function of the NF-κB pathway and inhibit or promote inflammatory responses may be a novel approach to ameliorate organ ischemia-reperfusion injury.

#### 2.2.1. Heart

When separating rat myocardial tissue to establish an I/R injury model in vivo, researchers found that M2-macrophage-derived exosomes (M2-exos) carry miRNA-148a into myocardial cells and bind to thioredoxin-interacting protein (TXNIP), which can inhibit the NF-κB signaling pathway and reduce myocardial damage caused by inflammation [46].

#### 2.2.2. Brain

The exosome-mediated NF-κB signaling pathway has significant advantages in improving cerebral infarction and inhibiting neuronal pyroptosis. Studies have shown that melatonin-treated P-exos have a significant effect on the treatment of inflammation after ischemia, which can reduce the pyrodeath of microglia and neurons by regulating the NF-κB signaling pathway, thus reducing the size of cerebral infarction [47]. On the other hand, extracellular vesicles derived from mesenchymal stem cells (MSCs) have shown broad potential for treating brain dysfunction and protecting nerves through nerve remodeling and angiogenesis [93]. MSCs-EVs can reduce cortical neuron apoptosis and improve cerebral infarction by down-regulating the phosphorylation level of the NF-κB signaling pathway [81]. Chinese herbal decoction can also affect the NF-κB pathway and improve cerebral ischemia-reperfusion injury. For example, atractylodes macrocephala inhibits the uptake of miRNA-200a-3p by astrocyte-derived exosomes, thereby enhancing the expression of sirtuin1 (SIRT1) in the cerebral cortex, binding to the p65 subunit of NF-κB and attenuating the inflammatory response in brain I/R injury [48].

#### 2.2.3. Intestines

Mesenteric lymph (ML) plays a crucial role in relieving intestinal inflammation. During intestinal ischemia, ML exosomes increase the concentration of lysophosphatidylcholine (LPC) in polyunsaturated fatty acids (PUFs), thereby activating the NF-κB signaling pathway and working with the active mediators in ML exosomes to regulate the ischaemic inflammatory response [49].

#### 2.2.4. Kidney

A number of studies have reported that exosomes have a vital function in renal ischemia-reperfusion injury, all of which provides new ideas for the treatment of renal ischemia-reperfusion injury [94]. Kim et al. compared several mice injected with exosome inhibitors or exosomes, showing decreased NF-κB activity and reduced apoptosis in the kidney after ischemia in the exosome-injected group [95]. In the established rat model of renal ischemia-reperfusion injury, exosomes derived from human urine-derived stem cells (HUSCs) carry miRNA-146a-5p to act on the mRNA of interleukin-1 receptor-associated kinase 1 (IRAK1), thereby inhibiting the activation of NF-κB signaling to maintain basic renal function [50]. In addition, Pan et al.’s study proved that remote ischemic preconditioning (rIPC) can up-regulate miRNA-21 expression at the injured site in a hypoxia-induced factor 1α (HIF-1α)-dependent manner and reduce the accumulation of systemic inflammatory cytokines. Serum exosomes can transport miRNA-21 to renal tubular epithelial cells (RTEC) to regulate the NF-κB pathway for anti-inflammatory and anti-apoptotic effects [51].

### 2.3. Nrf2 Signaling Pathway

The nuclear factor erythroid 2-related factor 2 (Nrf2) belongs to the Cap-n-Coll family of basic leucine zipper proteins. It is an important transcription factor regulating the cellular oxidative stress response as well as a central regulator of cellular redox homeostasis [96]. The imbalance between free radicals and antioxidants in the body may lead to excessive production of reactive oxygen species (ROS), which, in turn, aggravates cell damage and inflammation in patients with ischemia-reperfusion injury [97]. This pathway can regulate the expression of antioxidant proteins, reduce the damage of reactive oxygen species and electrophilic reagents to cells, and maintain the body’s REDOX homeostasis, which is considered to be the most promising signaling pathway in the clinical improvement of ischemia-reperfusion injury [98].

#### 2.3.1. Brain

Exosomes can protect and nourish neurons by activating the Nrf2 signaling pathway to alleviate cerebral ischemia-reperfusion injury. Bai et al. found that uric acid may play a neuroprotective role by activating the Nrf2 pathway to regulate neurotrophic factors in the cerebral cortex after focal cerebral I/R in rats [99]. Therefore, reducing the level of reactive oxygen species and down-regulating the expression of inflammatory factors by the antioxidant Nrf2 can reduce oxidative-stress-induced injury, which can be used for the treatment of organ ischemia-reperfusion injury [100]. Increasing the nuclear translocation of Nrf2 provides a new idea for the treatment of I/R injury. Exosomes targeted by neurons pass the BBB to initiate the Nrf2/Heme oxygenase 1 (HO-1) pathway, which induces an increase in nuclear Nrf2 level while activating SOD to remove superoxide radicals. Thus, the infarct size can be reduced and targeted therapy can be provided for cerebral ischemia-reperfusion injury [52]. Similarly, Evs secreted by human neural stem cells (HNSCs) overexpressing miRNA-133b enhance the nuclear translocation of Nrf2 by up-regulating the expression of antioxidant-related enzymes, while promoting the activation of downstream antioxidant enzymes to scavenge excessive free radicals, resulting in neuroregeneration and endogenous angiogenesis [53]. The establishment of a glucose deprivation/reperfusion (GD/R) cell model has important research significance for ischemia-reperfusion injury. In an in vitro oxygen/GD and reoxygenation/GD model, M2-polarized macrophages activate the Nrf2/HO-1 signaling pathway by releasing exosomes, improve the level of superoxide dismutase, and inhibit the production of reactive oxygen species, so as to alleviate the neuronal damage caused by oxidative stress, providing a new idea for the treatment of ischemic stroke [54].

#### 2.3.2. Spinal Cord

Restoring mitochondrial function is essential for the treatment of I/R injury. Extracellular vesicles (EVs) derived from melatonin-pretreated MSCs promote motor behavior recovery by inducing M1 to M2 polarization through the Nrf2 pathway. At the same time, the mitochondrial regulatory function of MSCs mRNA is enhanced to ameliorate spinal cord injury, which represents a promising strategy for the treatment of spinal cord injury [101].

#### 2.3.3. Kidney

The MSCs-Evs mediated Nrf2 signaling pathway has bright prospects for the treatment of renal ischemia-reperfusion injury. MSCs-Evs up-regulate the expression of miRNA-200a-3p through the Keap1-Nrf2 signaling pathway in animal models of acute renal injury with I/R, effectively alleviating mitochondrial damage in proximal RTECs and restoring mitochondrial antioxidant function to protect the kidney [55]. On the other hand, MSCs-EVs protect the kidney from ischemia-reperfusion injury in vivo and in vitro by activating the Nrf2/HO-1 signaling pathway to reduce creatinine and urea nitrogen levels and inhibit the expression of inflammatory factors and superoxides [56].

### 2.4. PTEN Signaling Pathway

Phosphatase and Tensin Homolog deleted on Chromosome 10 (PTEN) is a tumor suppressor with growth and survival regulatory functions. In recent years, it has been found that PTEN can participate in cell proliferation, energy metabolism, and other cellular activities as a metabolic regulator [102]. PTEN can inhibit the activation of downstream proteins of the PI3K pathway by reducing the level of phosphatidylinositol 3-phosphate (PIP3) in cells. It plays an important role in inhibiting many biological processes such as inflammation and apoptosis caused by I/R injury [103]. Studies have shown that mitochondria play an important role between the PTEN signaling pathway and I/R injury. The increase in mitochondrial autophagy mediated by PTEN-induced enzymes can reduce I/R injury and induce an inflammatory response to a certain extent, providing a new idea for the treatment of organ ischemia-reperfusion injury [104]. A variety of miRNAs carried by different exosomes derived from various stem cells can target the expression of the PTEN signaling pathway, which provides a variety of methods for the treatment of organ ischemia-reperfusion injury.

#### 2.4.1. Heart

Cardiac ischemia-reperfusion injury often leads to the apoptosis of a large number of cardiomyocytes and many cascade reactions, including apoptosis, mitochondrial dysfunction, and excessive autophagy [105]. The miRNA carried by exosomes plays an important role in inhibiting cardiomyocyte apoptosis and improving myocardial ischemia-reperfusion injury. Under the action of miRNA-29c carried by HBMSCs-exos, the effect of the PTEN/Akt/mTOR pathway on the excessive autophagy of cells is attenuated and the heart is protected from excessive oxidative stress damage [57]. The fluorescent-labeled in vitro injection of MSCs-EVs showed that exogenous miRNA-21 carried by MSCs-EVs was efficiently internalized into myocardial cells and silenced the expression of PTEN, inhibiting cell apoptosis, relieving inflammation, and improving cardiac function [58].

#### 2.4.2. Kidney

HUSCs-exos play an important role in exposed H/R-induced human tubular endothelial cells. HUSCs-exos are rich in miRNA-216a-5p, which can target the regulation of PTEN level and stimulate the phosphorylation of Akt, so as to protect cells and inhibit inflammatory response [59].

#### 2.4.3. Lung

Exosomes secreted by MSCs have a protective effect on oxidative-stress-induced cells. In the established mouse I/R model and in vitro H/R model using primary mouse lung endothelial cells, it is found that the occurrence of pulmonary edema and pulmonary dysfunction is significantly reduced in the model treated with MSCs-exos, and MSCs-exos inhibit the PTEN signaling pathway via miRNA-21-5p, thereby improving apoptosis induced by ischemia-reperfusion [60].

#### 2.4.4. Intestines

The PTEN pathway related to exosomes is also widely used in intestinal ischemia-reperfusion injury. Zhang et al. showed that HBMSCs-exos can also regulate the expression of the PTEN signaling pathway through miRNA-144-3p, reduce intestinal apoptosis, and effectively alleviate intestinal oxidative stress and intestinal pathological damage [61].

#### 2.4.5. Liver

HBMSCs-exos also play an important role in liver I/R injury. After injecting HBMSCs-exos and miRNA-25b-3p into the tail vein of mice with liver injury, the expression of PTEN was significantly decreased and the symptoms of liver cell necrosis were alleviated [62].

### 2.5. Wnt Signaling Pathway

The wingless/Integrated (Wnt) signaling pathway is highly conserved in invertebrates and vertebrates [106]. It plays a vital role in cell growth, proliferation, differentiation, and homeostasis [107]. The Wnt signaling pathway is a complex regulatory system that consists of three branches: the classical Wnt signaling pathway, the Wnt/planar cell polarity (PCP) pathway, and the Wnt/Ca^2+^ pathway. Among them, the classical Wnt signaling pathway activated by β-catenin is most closely related to the treatment of ischemia-reperfusion injury [108]. Lithium has been shown to alleviate BBB destruction after cerebral I/R in mice by up-regulating endothelial Wnt/β-catenin signaling [109]. Clearly, this pathway plays a significant role in the inhibition of apoptosis, which is involved in the regulation of protein levels, cell migration, and other processes closely related to the treatment of organ I/R injury [110].

#### 2.5.1. Brain

The Wnt signaling pathway plays an important role in the regeneration of cerebral endothelial blood vessels and the inhibition of inflammatory response. The Wnt pathway mediated by various exosomes provides new therapeutic ideas for ischemia-reperfusion injury. A high expression of chemokine receptor type 4 (CXCR4) in HBMSCs-exos induces the proliferation and migration of bend.3 cells through the Wnt pathway. At the same time, the expression level of anti-apoptotic proteins is enhanced, which promotes vascular formation and re-epithelialization, providing promising strategies for the treatment of stroke diseases [63]. On the other hand, the Wnt signaling pathway can effectively reduce the expression of pro-inflammatory cytokines and play an anti-apoptotic role [111].

#### 2.5.2. Heart

In cardiomyocytes, exosomes derived from ADSCs exert anti-apoptotic effects in H/R-induced H9C2 cell models by activating the Wnt/β-catenin signaling pathway and reducing lactate dehydrogenase (LD) and cardiac troponin I levels, which effectively ameliorates myocardial infarction caused by I/R injury [64]. In addition, HBMSCs-exo can effectively prevent the ischemia-reperfusion injury of myocardial cells. They carry miRNA-149 into myocardial tissue to regulate Faslg expression, activate Wnt/β-catenin signaling, and reduce the cell production of reactive oxygen species, thus achieving the purpose of heart protection [65]. Evs derived from anoxic human MSCs overexpressed miR-26a, significantly inhibiting the expression of glycogen synthetase kinase-3 (GSK-3) by the Wnt pathway and promoting myocardial cell survival. This can effectively reduce the degree of infarction expansion and provide a new beneficial strategy for protecting myocardial injury [66].

#### 2.5.3. Liver

In rats with hepatic I/R injury, ADSCs-exos induce M1–M2 microglia polarization by activating the Wnt signaling pathway, alleviate hepatic ischemia-reperfusion injury, inhibit the expression of pro-inflammatory cytokines, and eliminate inflammation [112].

### 2.6. MAPK Signaling Pathway

Mitogen-activated protein kinase (MAPK) is a group of serine/threonine protein kinases that can be activated by different extracellular stimulation. It is involved in many biological processes such as apoptosis, hormone signal transduction, and the regulation of inflammatory factors [113]. It has been confirmed that MAPK genes can be divided into three major subfamilies, namely extracellular-signal-regulated kinases (ERKs), the p38 MAPKs, and Jun N-terminal kinases (JNKs) [114].

The MAPK pathway is involved in a variety of biological processes, such as protecting nerve cells and inhibiting inflammatory response, which is of great significance for ameliorating brain I/R injury [115]. Huang et al. found that curcumin can inhibit the MAPK/p38 pathway, effectively reduce NLR family pyrin domain containing 1 (NLRP1)-dependent neuronal pyroptosis, and thus play a neuroprotective role in cerebral I/R injury [116]. In addition, studies have shown that the inflammation of ischemia-reperfusion injury can be alleviated by reducing the expression levels of phosphorylated p38, ERK, and JNK [117].

#### 2.6.1. Brain

Exosomes and certain miRNAs are also critical for mitigating I/R injury. In mouse models of middle cerebral artery occlusion (MCAO), stem-cell-derived EVs demonstrated remarkable anti-inflammatory effects, with seven miRNAs packaged within them capable of inhibiting the MAPK pathways to reduce inflammation [67]. In addition, Wu et al. found that astrocyte-derived exosomes can carry miRNA-34c to act on toll-like receptors, inhibit the MAPK pathway, and relieve nerve damage caused by I/R, which provides a new idea for the treatment of brain I/R injury [68].

#### 2.6.2. Liver

ADSCs-exos have a high application value in liver ischemia-reperfusion injury. ADSCs-exos are used to pretreat the liver ischemia-reperfusion injury model, playing an anti-apoptotic role by up-regulating the ERK1/2 pathway. This approach significantly reduces liver tissue necrosis and apoptosis caused by ischemia-reperfusion injury [69]. ADSCs-exos can also alleviate liver I/R injury. They carry miRNA-183 to act on arachidonic acid 5-lipoxygenase (ALOX5), inhibit the MAPK pathway through the miRNA-183/ALOX5 axis, induce the proliferation of human hepatocytes, and inhibit apoptosis [70].

#### 2.6.3. Kidney

Studies have confirmed that human amniotic epithelial cells (HAECs) and their derived exosomes can effectively prevent ischemia-reperfusion-induced acute kidney injury. HAECs and exosomes can reduce renal dysfunction and pathological injury by inhibiting the MAPK and caspase signaling pathways [71].

#### 2.6.4. Heart

Zhao et al. found through experiments that small EVs secreted by basic brown adipose tissue (BAT), which mediates cardiac protection, inhibit the activation of related MAPK pathways by transporting the basic components of cardiac protection, such as miRNA-125b-5p and miRNA-128-3p, to myocardial tissue, thus achieving the purpose of protecting the heart [72].

### 2.7. Toll-Like Receptor-Mediated Signaling Pathway

Toll-like receptors (TLRs) are well known for their general effects in non-specific immunity. Initially, TLRs were discovered in developmental studies, and as research progressed, TLRS were known to regulate cellular communication and signaling in synapses [118]. Expressed TLRs can be released by host cells and activate various intracellular pathways, leading to the production of pro-inflammatory cytokines and the expression of costimulatory molecules, so as to prevent pathogen invasion and damage to the human body [119].

The Toll-like receptor-mediated signaling pathway has been identified as one of the signaling pathways involved in the inflammatory response in ischemia-reperfusion injury [120]. In the treatment of I/R injury, various active substances carried by exosomes from various cell sources can be combined with TLR to alleviate I/R injury. TLR2 has been reported to promote the release of interleukin-10 from macrophages to regulate the levels of inflammatory cytokines in I/R injury [121]. Furthermore, TLR9, as a key receptor for unmethylated CpG motifs in mitochondrial DNA, regulates the occurrence of inflammatory responses [122]. Blocking the TLR-mediated signaling pathway can improve myocardial I/R injury and reduce infarct size, which may be a new target for the treatment of organ ischemia-reperfusion injury [123].

#### 2.7.1. Brain

The presence of the BBB makes it difficult for drugs to act on the site of cerebral ischemia reperfusion injury, and Shireen et al. found that P-exos have certain advantages in this respect [124]. P-exo is an effective carrier for carrying heat shock protein 70 (HSP70) and delivering it to the brain. It interacts with TLR4 to inhibit ROS production, thereby reducing mitochondrial apoptosis and reducing ischemic brain damage [73]. In the established I/R mouse model, miRNA-26b-5p carried by HUCMC-exos can effectively inhibit the TLR pathway and attenuate the M1 polarization of microglia to alleviate brain nerve crush [74]. Studies have shown that a low expression of miRNA-150-5p is one of the causes of high mortality in patients with ischemic stroke. In the establishment of a middle cerebral artery occlusion rat model, miRNA-150-5p transported by the exosomes of HBMSCs can bind to TLR5 and silence its expression, so as to prevent nerve apoptosis, inhibit the level of inflammatory factors, and improve the neurological function of MCAO rats [75].

#### 2.7.2. Heart

The Toll-like receptor-mediated signaling pathway can reverse myocardial ischemia-reperfusion injury by reducing the content of TLR4. HBMSC-derived exosomes can also act on TLR4 through miRNA-98-5p. In the myocardial tissue of mice with I/R injury, miRNA-98-5p targeted binding with TLR4 promotes the protective function of the heart, inhibits the myocardial enzyme level and oxidative stress response in the myocardial tissue, and improves myocardial ischemia reperfusion injury [76]. In addition, Zheng’s team showed that EVs derived from human umbilical vascular endothelial cells (HUVECs) can enhance the expression of miRNA-129, allowing it to bind to the 3’ untranslated region of TLR4 more rapidly and down-regulate the expression level of TLR4. Thus, it can degrade the pro-inflammatory factors produced in cells and relieve myocardial fibrosis, which provides potential for the treatment of cardiac I/R injury [77].

#### 2.7.3. Intestines

A research team used the extracellular vesicles of Akkermansia muciniphila bacteria to improve both intestinal and blood–brain barrier functions, alleviating cognitive dysfunction in mice with intestinal ischemia-reperfusion, which provides new ideas for the treatment of I/R injury [125].

### 2.8. AMPK Signaling Pathway

AMPK (Adenosine 5′-monophosphate activated protein kinase) is a key molecule in the regulation of biological energy metabolism. It is expressed in a variety of metabolically related organs and can be activated by various types of stimulation, including cell movement, hormones, and substances that affect cell metabolism [126]. The AMPK pathway is a pleiotropic signaling pathway involved in many processes, such as regulating lipid metabolism and glucose metabolism, maintaining mitochondrial function stability, and cell energy homeostasis [127]. In addition, it positively regulates intracellular ATP supply, reduces energy consumption, and enhances autophagy, which is considered to be a key therapeutic target for the treatment of organ ischemia-reperfusion injury [128].

AMPK activation reduces oxidant-induced injury in I/R Injury by promoting autophagy, inhibiting apoptosis, and up-regulating the antioxidant enzyme system [129]. HBMSC-derived exosomes decrease caspase 1 and interleukin-1β levels and increase the number of autophagosomes and autolysosomes by activating the AMPK pathway. At the same time, the pyrodeath process mediated by leucine-rich NLRP3 inflammasome is weakened, thereby protecting PC12 cells from ischemia-reperfusion injury [78].

#### 2.8.1. Heart

Exosomes from different sources play an active role in the remodeling of cardiomyocytes by activating the AMPK pathway. Studies have demonstrated the beneficial effects of MSC-derived exosomes on myocardial remodeling. Liu et al. demonstrated that exosomes derived from MSCs reduced H_2_O_2_-induced ROS production and apoptosis by regulating the AMPK pathway, enhancing autophagy while significantly reducing the size of myocardial infarction, thereby improving cardiac function [79]. In I/R mice, LINC00174, which is highly expressed in exosomes from aortic endothelial cells, activates the AMPK pathway to improve cardiomyocyte function, down-regulates myocardin expression, and ameliorates myocardial injury by promoting autophagy and apoptosis [80].

#### 2.8.2. Brain

MSCs-Evs have shown broad potential for treating brain dysfunction through neuroangiogenesis. MSCs-EVs can reduce the level of oxidative stress after middle cerebral artery occlusion MCAO in rats and reduce the release of inflammatory factors and energy consumption after cerebral ischemia by significantly up-regulating the phosphorylation level of the AMPK signaling pathway, which provides an effective strategy for the treatment of cerebral infarction [81]. By inducing the overexpression of miRNA-369-3p in the exosomes of HBMSCs, human growth differentiation factor 7 down-regulates the phosphodiesterase levels in primary neurons, thereby activating the AMPK pathway, effectively antagonizing I/R-induced inflammation, oxidative stress, and nerve damage [82].

**Figure 4:**
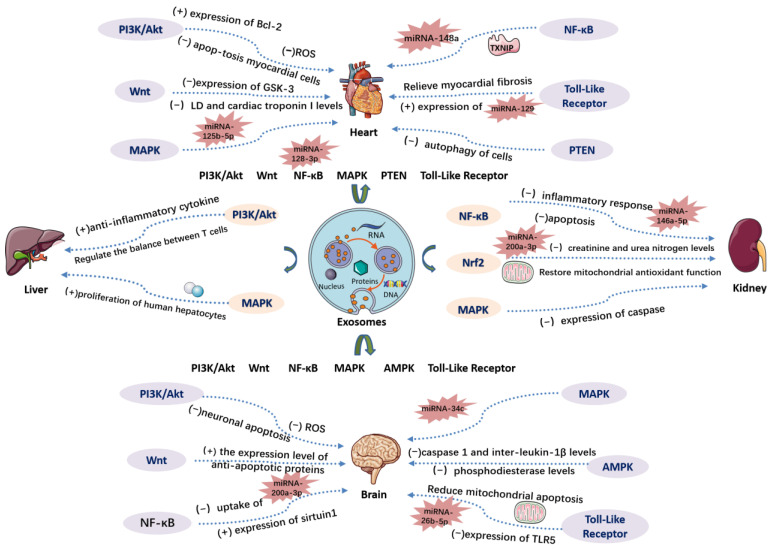
This figure shows the mechanism of action of the exosomes described in the manuscript on the heart, brain, kidney, liver, and other major organs with ischemia-reperfusion injury by mediating PI3K/Akt, PTEN, Nrf2, MAPK, Wnt, and other signaling pathways.

In summary, the main mechanism of exosomes mediating the above eight signaling pathways in the treatment of ischemia-reperfusion injured organs is shown in Figure 4.

### 2.9. Cross-Talk between Different Signaling Pathways

In addition to treating organ I/R damage through the above exosome-mediated signaling pathways, exosomes can also act on I/R damage by regulating the cross-talk between different signaling pathways.

#### 2.9.1. Heart

The P-exos isolated after rIPC are rich in miRNA-126a-3p, which a show significant cardiac protective effect. By enhancing Akt and Erk1/2 phosphorylation, the reperfusion injury salvage kinase (RISK) pathway is activated and the activation of apoptotic protein Caspase-3 is inhibited, thereby reducing myocardial I/R injury [130]. In the I/R injury model established in rats, the abundant miRNA-455-3p in HBMSCs-exo inhibits the expression of the Mitogen/Extracellular regulatory protein kinase 1(MEKK1)-Mitogen activated protein kinase kinase 4 (MKK4)-JNK signaling pathway, reduces cell apoptosis, and increases myocardial cell vitality, which has a protective effect on myocardial injury by I/R and also provides a new idea for the treatment of myocardial infarction [131]. In addition, Yang et al. demonstrated that miRNA-140-3p carried by exosomes derived from endothelial colony-forming cells can indirectly inhibit the expression of inflammatory factors by inhibiting the activation of the PTEN pathway and promoting the phosphorylation of the PI3K/Akt signaling pathway, thereby mediating angiogenesis and myocardial injury repair [132].

#### 2.9.2. Brain

Exosomes mediate the interaction between different signaling pathways, which also brings certain hope for the treatment of brain ischemia-reperfusion injury. Astrocyte-derived exosomes promote the proliferation of N2a cells and transport miRNA-34c to TLR7 by targeting them. This down-regulates the NF-κB/MAPK pathway and prevents the overactivation of pro-inflammatory cytokines, thereby reducing I/R-induced nerve damage [68].

#### 2.9.3. Kidney

On the other hand, Douvris’ team found that, under the influence of exosomes carried miRNA-486-5p, cross-talk between PTEN and PI3K/Akt not only reduces myocardial cell apoptosis and heart infarct size, but also lessens the amount of cell necrosis caused by renal I/R injury and can alleviate renal failure [133]. In addition, the hypoxia-induced release of EVs from RTECs has a more significant protective effect against renal I/R injury in the interaction of the HIF-1α/Rab22 pathway [134].

#### 2.9.4. Liver

HUCMCs show the potential of modulating the immune response and promoting cell regeneration, which can precisely be used as a treatment for the inflammatory response and apoptosis caused by I/R injury. HUCMCs-EVs can regulate the T cell level in the liver through Ca^2+^/calcineurin/nuclear factor activating the T cell 1(NFAT1) signaling pathway, and interfere with the initiation of inflammatory response in liver I/R injury so as to treat liver I/R injury [135].

The combined action of different signaling pathways regulated by exosomes can effectively treat organ I/R injury to a certain extent, and also provide ideas and directions for the future treatment of I/R injury.

## 3. Conclusions and Future Aspects

In this review, the effects of exosomes from different sources and the mechanisms of their mediated signaling pathways were elucidated. It was shown that exosomes can regulate signaling pathways such as PI3K/Akt, NF-κB, Nrf2, PTEN, Wnt, MAPK, toll-like receptor, and AMPK to remove free radicals, reduce inflammation and intracellular calcium ion load, and relieve oxidative stress to treat I/R injury. Among them, the most promising signaling pathway might be Nrf2, as it is the most important transcription factor regulating cellular oxidative stress response and can regulate the balance between free radicals and antioxidants in the body [96]. In particular, MSC-derived exosomes have shown an improved therapeutic efficacy compared to MSCs, suggesting that exosomes may be a more effective cell-free therapy. However, not all types of exosomes are beneficial for physiological activities. For example, the secretion of non-degraded autophagosomes in the form of exosomes leads to an inflammatory cascade that further destroys mitochondria, overproducing ROS and exacerbating inflammation during cerebral ischemia-reperfusion [136]. In addition, tumor-derived exosomes are emerging mediators of cancer cachexia, while exosomes from visceral adipose tissue increase the severity of colitis through pro-inflammatory miRNAs [137,138]. Hence, the types and sources of exosomes should be carefully selected for the application of exosomes to treat I/R injury and other diseases.

Despite the numerous evidence that exosomes can be used to treat organ I/R injury, the application of exosomes clinically has been hindered by many aspects, including the lack of uniform methods for exosome isolation and characterization, missing knowledge of the exact interactions between exosomes with target cells and organs, difficulties in the mass production and storage of exosomes, and so on [139]. In the study by Qiu et al., the clinical use of MSC-exos in alleviating myocardial I/R damage was hindered by low cell implantation rates and uncontrolled exosome content [30]. The different separation protocols may result in different subpopulations of exosomes with different miRNAs, proteins, functions, and non-vesicular macromolecules [140], making it difficult to recognize which molecules in exosomes are responsible for the observed effects. In addition, the complexity of signal transduction and the cross-talk between different pathways also increase the difficulty in understanding the precise mechanism of exosomes in treating diseases [141]. Therefore, prospective studies should focus on the standardization of these isolation and purification processes and a detailed exploration of different subgroups of exosomes, so as to clarify their exact mechanism and pave the way for the efficient clinical application.

## Figures and Tables

**Table 1:** The effect of different exosomes on the treatment of organ ischemia-reperfusion injury through different signaling pathways.

The Cellular Origin of Exosomes	Experimental Models		Mechanism/Results	Signaling Pathway	Ref.
	In Vivo	In Vitro			
Stem-cell-derived	Focal cerebral I/R model	Neurons	Reduce the expression of inflammatory cytokines; inhibit neuronal apoptosis	PI3K/Akt	[39]
Human bone marrow mesenchymal stem cells	Myocardial I/R model	Cardiomyocytes	Reduce HCP5 expression	PI3K/Akt	[40]
Human bone marrow mesenchymal stem cells	Myocardial I/R model	H9C2s	Promote the proliferation of H9C2 cells; inhibit the apoptosis myocardial cells	PI3K/Akt	[41]
Plasma	Myocardial I/R model	-	Up-regulate the expression of Bcl-2; reduce the expression of inflammatory cytokines	PI3K/Akt	[42]
Bone-marrow-derived dendritic cells	Hepatocyte H/R model	T cells	Enhance anti-inflammatory cytokine secretion; regulate the balance between different T cells	PI3K/Akt	[43]
Adipose-derived stromal cells	-	Spermatogenic cells	Induce spermatocyte proliferation and migration; reduce oxidative stress and inflammatory factors	PI3K/Akt	[44]
Human umbilical cord mesenchymal cells	Myocardial H/R model	H9C2s	Inhibit the activation of caspase-3 and ER stress markers expression; reduce cell apoptosis	PI3K/Akt	[45]
M2-macrophage-derived	Myocardial I/R model	Cardiomyocytes	Carry miRNA-148a and bind to TXNIP; relieve inflammation	NF-κB	[46]
Plasma	Focal cerebral I/R model	Neurons	Reduce the pyrodeath of microglia and neurons; reduce the size of cerebral infarction	NF-κB	[47]
Astrocyte	Brain I/R model	-	Inhibit the uptake of miR-200a-3p by exosomes; enhance the expression of sirtuin1 in cerebral cortex	NF-κB	[48]
Mesenteric lymph	-	Mesenteric lymphocytes	Increase the concentration of LPC in PUFs	NF-κB	[49]
Human urine-derived stem cells	Renal I/R model	Renal tubular cells	Carry miRNA-146a-5p to act on the mRNA of IRAK1	NF-κB	[50]
Serum	-	Renal tubular epithelial cells	Inhibit inflammatory response and reduce apoptosis	NF-κB	[51]
Neuron	Middle cerebral artery occlusion/reperfusion (MCAO/R) model	Neurons	Remove superoxide radicals	Nrf2	[52]
Human neural stem cells	Brain I/R model	Neurons	Up-regulate the expression of vasotropic factors; promote the proliferation and migration of vascular endothelial cells	Nrf2	[53]
M2-polarized macrophages	Glucose deprivation/reperfusion (GD/R) cell model	Neurons	Improve the level of superoxide dismutase; inhibit the production of reactive oxygen	Nrf2	[54]
Mesenchymal stem cells	Renal I/R model	Renal tubular epithelial cells	Up-regulate the expression of miRNA-200a-3p; restore mitochondrial antioxidant function	Nrf2	[55]
Mesenchymal stem cells	Renal I/R model	Renal tubular epithelial cells	Reduce creatinine and urea nitrogen levels; inhibit the expression of inflammatory factors	Nrf2	[56]
Human bone marrow mesenchymal stem cells	Myocardial I/R model	-	Attenuate autophagy of cells; inhibit oxidative stress	PTEN	[57]
Mesenchymal stem cells	-	Cardiomyocytes	Inhibit cell apoptosis; relieve inflammation	PTEN	[58]
Human urine-derived stem cells	Renal I/R model	Renal tubular epithelial cells	Stimulate phosphorylation of Akt; inhibit inflammatory response	PTEN	[59]
Mesenchymal stem cells	Lung I/R model	Lung endothelial cells	Reduce the occurrence of pulmonary edema and pulmonary dysfunction	PTEN	[60]
Human bone marrow mesenchymal stem cells	GD/R cell model	Intestinal cells	Carry miRNA-144-3p; reduce the apoptosis of intestinal cells	PTEN	[61]
Human bone marrow mesenchymal stem cells	Liver I/R model	-	Reduce apoptosis, oxidative stress, and DNA damage	PTEN	[62]
Human bone marrow mesenchymal stem cells	MCAO/R model	Cerebral endothelial cells	Induce the proliferation and migration of bend.3 cells; enhance the expression level of anti-apoptotic proteins	Wnt	[63]
Adipose-derived stromal cells	Myocardial I/R model	H9C2s	Exert anti-apoptotic effects; reduce LD and cardiac troponin I levels	Wnt	[64]
Human bone marrow mesenchymal stem cells	-	H9C2s	Regulate Faslg expression; reduce cell production of reactive oxygen species	Wnt	[65]
Mesenchymal stem cells	Myocardial I/R model	Cardiomyocytes	Inhibit the expression of GSK-3; reduce the degree of infarction expansion	Wnt	[66]
Stem-cell-derived	MCAO/R model	Neural progenitor cells	Package seven miRNAs; reduce inflammation	MAPK	[67]
Astrocytes	Brain I/R model	Neurons	Carry miRNA-34c to act on toll-like receptors	MAPK	[68]
Adipose-derived stromal cells	Lung I/R model	-	Play an anti-apoptotic role; reduces liver tissue necrosis and apoptosis	MAPK	[69]
Adipose-derived stromal cells	Liver I/R model	Human hepatocytes	Induce proliferation of human hepatocytes and inhibit apoptosis	MAPK	[70]
Human amniotic epithelial cells	Renal I/R model	-	Inhibit the expression of caspase; reduce renal dysfunction	MAPK	[71]
Brown adipose tissue	Myocardial I/R model	Cardiomyocytes	Transport miRNA-125b-5p and miRNA-128-3p to myocardial tissue	MAPK	[72]
Plasma	Brain I/R model	-	Deliver HSP70 to the brain; inhibit ROS production; reduce mitochondrial apoptosis	Toll-like receptor mediated	[73]
Human umbilical cord mesenchymal cells	Brain I/R model	Microglial cells	Carry miRNA-26b-5p; attenuate the M1 polarization of microglia	Toll-like receptor mediated	[74]
Human bone marrow mesenchymal stem cells	MCAO/R model	-	Silence TLR5 expression; inhibit the level of inflammatory factors	Toll-like receptor mediated	[75]
Human bone marrow mesenchymal stem cells	Myocardial I/R model	Cardiomyocytes	Inhibit the myocardial enzyme level and oxidative stress response	Toll-like receptor mediated	[76]
Human umbilical vascular endothelial cells	Myocardial I/R model	Cardiomyocytes	Enhance the expression of miRNA-129; degrade the pro-inflammatory factors; relieve myocardial fibrosis	Toll-like receptor mediated	[77]
Human bone marrow mesenchymal stem cells	Brain I/R model	-	Decrease caspase 1 and interleukin-1β levels; alleviate neuronal apoptosis	AMPK	[78]
Mesenchymal stem cells	Myocardial I/R model	H9C2s	Reduce ROS production and apoptosis; enhance autophagy	AMPK	[79]
Aortic endothelial cells	Myocardial I/R model	Cardiomyocytes	Down-regulate myocardin expression; promote autophagy and apoptosis	AMPK	[80]
Mesenchymal stem cells	MCAO/R model	-	Reduce the level of oxidative stress; decrease the release of inflammatory factors	AMPK	[81]
Human bone marrow mesenchymal stem cells	Brain I/R model	Neuron	Down-regulate phosphodiesterase levels in primary neurons	AMPK	[82]

## Abbreviations

ADSCs	Adipose-derived stromal cells
AKT	Serine/threonine kinase or protein kinase B (PKB)
AMPK	Adenosine 5′-monophosphate activated protein kinase
BAT	Brown adipose tissue
BBB	Blood-brain barrier
Bcl-2	B-cell lymphoma-2
BMDCs	Bone-marrow-derived dendritic cells
CXCR4	Chemokine receptor type 4
ER	Endoplasmic reticulum
ERKs	Extracellular signal regulated kinases
EVs	Extracellular vesicles
GD/R	Glucose deprivation/reperfusion
GSK-3	Glycogen synthetase kinase-3
HAECs	Human amniotic epithelial cells
HBMSCs	Human bone marrow mesenchymal stem cells
HCP5	HLA complex P5
HDPSCs	Human dental pulp stem cells
HIF-1α	Hypoxia-induced factor 1α
HNSCs	Human neural stem cells
HO-1	Heme oxygenase 1
H/R	Hypoxia/Reoxygenation
HSP70	Heat shock protein 70
HUCMCs	Human umbilical cord mesenchymal cell
HUSCs	Human urine-derived stem cells
HUVECs	Human umbilical vascular endothelial cells
IPC	Ischemic preconditioning
I/R	Ischemia-reperfusion
IRAK1	Interleukin-1 receptor-associated kinase 1
JNKs	Jun N-terminal kinases
LD	Lactate dehydrogenase
LPC	Lysophosphatidylcholine
MAPK	Mitogen-activated protein kinase
MCAO	Middle cerebral artery occlusion
MDA	Malondialdehyde
MEKK1	Mitogen/Extracellular regulatory protein kinase 1
MIRI	Myocardial ischemia-reperfusion injury
MKK4	Mitogen activated protein kinase kinase 4
ML	Mesenteric lymph
MSCs	Mesenchymal stem cells
MSCs-EVs	Extracellular vesicles derived from MSCs
M2-exos	M2 macrophage derived exosomes
NFAT1	Nuclear factor of activating T cell 1
NF-κB	Nuclear factor kappa-B
NLRP1	NLR family pyrin domain containing 1
Nrf2	The nuclear factor erythroid 2-related factor 2
PCP	The Wnt/planar cell polarity
P-exos	Plasma exosomes
PI3K	Phosphatidylinositol-3-kinase
PIP3	Phosphatidylinositol 3-phosphate
PTEN	Phosphatase and Tensin Homolog deleted on Chromosome 10
PUFs	Polyunsaturated fatty acids
RIPC	Remote ischemic preconditioning
RISK	reperfusion injury salvage kinase
ROS	Reactive oxygen species
SOD	Superoxide dismutase
TEC	Tubular epithelial cell
TLRs	Toll-like receptors
TXNIP	Thioredoxin interacting protein
Wnt	The wingless/Integrated

## References

[1] Liu Y., Li L., Wang Z., Zhang J., Zhou Z. (2023). Myocardial ischemia-reperfusion injury; Molecular mechanisms and prevention. Microvasc. Res..

[2] Shi Y.J., Sun L.L., Ji X., Shi R., Xu F., Gu J.H. (2021). Neuroprotective effects of oleanolic acid against cerebral ischemia-reperfusion injury in mice. Exp. Neurol..

[3] Zheng T., Jiang T., Huang Z., Ma H., Wang M. (2023). Role of traditional Chinese medicine monomers in cerebral ischemia/reperfusion injury: A review of the mechanism. Front. Pharmacol..

[4] Fan L., Zhou L. (2021). AG490 protects cerebral ischemia/reperfusion injury via inhibiting the JAK2/3 signaling pathway. Brain Behav..

[5] Ikhlas M., Atherton N.S. (2022). Vascular Reperfusion Injury. StatPearls.

[6] Fan Z., Cai L., Wang S., Wang J., Chen B. (2021). Baicalin Prevents Myocardial Ischemia/Reperfusion Injury Through Inhibiting ACSL4 Mediated Ferroptosis. Front. Pharmacol..

[7] Jiménez-Castro M.B., Cornide-Petronio M.E., Gracia-Sancho J., Peralta C. (2019). Inflammasome-Mediated Inflammation in Liver Ischemia-Reperfusion Injury. Cells.

[8] Algoet M., Janssens S., Himmelreich U., Gsell W., Pusovnik M., Van den Eynde J., Oosterlinck W. (2023). Myocardial ischemia-reperfusion injury and the influence of inflammation. Trends Cardiovasc. Med..

[9] Nørgård M., Svenningsen P. (2023). Acute Kidney Injury by Ischemia/Reperfusion and Extracellular Vesicles. Int. J. Mol. Sci..

[10] Tao H., Dong L., Shan X., Li L., Chen H. (2023). MicroRNA-32-3p facilitates cerebral ischemia/reperfusion injury through inhibiting Cab39/AMPK. Int. Immunopharmacol..

[11] Wang R., Wang M., Zhou J., Dai Z., Sun G., Sun X. (2021). Calenduloside E suppresses calcium overload by promoting the interaction between L-type calcium channels and Bcl2-associated athanogene 3 to alleviate myocardial ischemia/reperfusion injury. J. Adv. Res..

[12] Ye J., Wang R., Wang M., Fu J., Zhang Q., Sun G., Sun X. (2021). Hydroxysafflor Yellow A Ameliorates Myocardial Ischemia/Reperfusion Injury by Suppressing Calcium Overload and Apoptosis. Oxidative Med. Cell. Longev..

[13] Zheng X., Li J., Fan Q., Zhao X., Chen K. (2021). Dexmedetomidine alleviates myocardial ischemia/reperfusion-induced injury and Ca(2+) overload via the microRNA-346-3p/CaMKIId axis. Int. J. Cardiol..

[14] Tu C.C., Wan B.Y., Zeng Y. (2020). STIM2 knockdown protects against ischemia/reperfusion injury through reducing mitochondrial calcium overload and preserving mitochondrial function. Life Sci..

[15] Li S., Chen J., Liu M., Chen Y., Wu Y., Li Q., Ma T., Gao J., Xia Y., Fan M. (2021). Protective effect of HINT2 on mitochondrial function via repressing MCU complex activation attenuates cardiac microvascular ischemia-reperfusion injury. Basic. Res. Cardiol..

[16] Francisco J., Del Re D.P. (2023). Inflammation in Myocardial Ischemia/Reperfusion Injury: Underlying Mechanisms and Therapeutic Potential. Antioxidants.

[17] Franke M., Bieber M., Kraft P., Weber A.N.R., Stoll G., Schuhmann M.K. (2021). The NLRP3 inflammasome drives inflammation in ischemia/reperfusion injury after transient middle cerebral artery occlusion in mice. Brain Behav. Immun..

[18] Huang J., Chen L., Yao Z.M., Sun X.R., Tong X.H., Dong S.Y. (2023). The role of mitochondrial dynamics in cerebral ischemia-reperfusion injury. Biomed. Pharmacother..

[19] Necsoiu C., Jordan B.S., Choi J.H., Moon J.J., Espinoza M.D., Gremmer B.J., Batchinsky A.I., Cancio L.C. (2021). Mitigating Ischemia-Reperfusion Injury Using a Bilobed Partial REBOA Catheter: Controlled Lower-Body Hypotension. Shock.

[20] Charles E.J., Tian Y., Zhang A., Wu D., Mehaffey J.H., Gigliotti J.C., Klibanov A.L., Kron I.L., Yang Z. (2021). Pulsed ultrasound attenuates the hyperglycemic exacerbation of myocardial ischemia-reperfusion injury. J. Thorac. Cardiovasc. Surg..

[21] Li X., Ou W., Xie M., Yang J., Li Q., Li T. (2023). Nanomedicine-Based Therapeutics for Myocardial Ischemic/Reperfusion Injury. Adv. Healthc. Mater..

[22] Liu J., Gu Y., Guo M., Ji X. (2021). Neuroprotective effects and mechanisms of ischemic/hypoxic preconditioning on neurological diseases. CNS Neurosci. Ther..

[23] Lou J., Wang X., Zhang H., Yu G., Ding J., Zhu X., Li Y., Wu Y., Xu H., Xu H. (2022). Inhibition of PLA2G4E/cPLA2 promotes survival of random skin flaps by alleviating Lysosomal membrane permeabilization-Induced necroptosis. Autophagy.

[24] Mu J., Li C., Shi Y., Liu G., Zou J., Zhang D.Y., Jiang C., Wang X., He L., Huang P. (2022). Protective effect of platinum nano-antioxidant and nitric oxide against hepatic ischemia-reperfusion injury. Nat. Commun..

[25] Eskla K.L., Vellama H., Tarve L., Eichelmann H., Jagomäe T., Porosk R., Oja V., Rämma H., Peet N., Laisk A. (2022). Hypothermia Alleviates Reductive Stress, a Root Cause of Ischemia Reperfusion Injury. Int. J. Mol. Sci..

[26] Ziegler M., Haigh K., Nguyen T., Wang X., Lim B., Yap M.L., Eddy E.M., Haigh J.J., Peter K. (2019). The pulmonary microvasculature entraps induced vascular progenitor cells (iVPCs) systemically delivered after cardiac ischemia-reperfusion injury: Indication for preservation of heart function via paracrine effects beyond engraftment. Microcirculation.

[27] Liu X., Zhang M., Liu H., Zhu R., He H., Zhou Y., Zhang Y., Li C., Liang D., Zeng Q. (2021). Bone marrow mesenchymal stem cell-derived exosomes attenuate cerebral ischemia-reperfusion injury-induced neuroinflammation and pyroptosis by modulating microglia M1/M2 phenotypes. Exp. Neurol..

[28] Hade M.D., Suire C.N., Suo Z. (2021). Mesenchymal Stem Cell-Derived Exosomes: Applications in Regenerative Medicine. Cells.

[29] Liang Y., Duan L., Lu J., Xia J. (2021). Engineering exosomes for targeted drug delivery. Theranostics.

[30] Gao L., Qiu F., Cao H., Li H., Dai G., Ma T., Gong Y., Luo W., Zhu D., Qiu Z. (2023). Therapeutic delivery of microRNA-125a-5p oligonucleotides improves recovery from myocardial ischemia/reperfusion injury in mice and swine. Theranostics.

[31] Chang C., Cai R.P., Su Y.M., Wu Q., Su Q. (2023). Mesenchymal Stem Cell-Derived Exosomal Noncoding RNAs as Alternative Treatments for Myocardial Ischemia-Reperfusion Injury: Current Status and Future Perspectives. J. Cardiovasc. Transl. Res..

[32] Wang S., Jun J., Cong L., Du L., Wang C. (2021). miR-328-3p, a Predictor of Stroke, Aggravates the Cerebral Ischemia-Reperfusion Injury. Int. J. Gen. Med..

[33] Hu S., Li Z., Shen D., Zhu D., Huang K., Su T., Dinh P.U., Cores J., Cheng K. (2021). Exosome-eluting stents for vascular healing after ischaemic injury. Nat. Biomed. Eng..

[34] Zhang R., Mao W., Niu L., Bao W., Wang Y., Wang Y., Zhu Y., Yang Z., Chen J., Dong J. (2023). NSC-derived exosomes enhance therapeutic effects of NSC transplantation on cerebral ischemia in mice. eLife.

[35] Mathew B., Ravindran S., Liu X., Torres L., Chennakesavalu M., Huang C.C., Feng L., Zelka R., Lopez J., Sharma M. (2019). Mesenchymal stem cell-derived extracellular vesicles and retinal ischemia-reperfusion. Biomaterials.

[36] Xin P., Xu X., Deng C., Liu S., Wang Y., Zhou X., Ma H., Wei D., Sun S. (2020). The role of JAK/STAT signaling pathway and its inhibitors in diseases. Int. Immunopharmacol..

[37] Liu X., Wang L., Ma C., Wang G., Zhang Y., Sun S. (2019). Exosomes derived from platelet-rich plasma present a novel potential in alleviating knee osteoarthritis by promoting proliferation and inhibiting apoptosis of chondrocyte via Wnt/β-catenin signaling pathway. J. Orthop. Surg. Res..

[38] Hu B., Tian T., Li X.T., Hao P.P., Liu W.C., Chen Y.G., Jiang T.Y., Chen P.S., Cheng Y., Xue F.S. (2023). Dexmedetomidine postconditioning attenuates myocardial ischemia/reperfusion injury by activating the Nrf2/Sirt3/SOD2 signaling pathway in the rats. Redox Rep. Commun. Free Radic. Res..

[39] Zhang Y., Yu J., Liu J., Liu H., Li J. (2021). Effects of stem cell-derived exosomes on neuronal apoptosis and inflammatory cytokines in rats with cerebral ischemia-reperfusion injury via PI3K/AKT pathway-mediated mitochondrial apoptosis. Immunopharmacol. Immunotoxicol..

[40] Li K.S., Bai Y., Li J., Li S.L., Pan J., Cheng Y.Q., Li K., Wang Z.G., Ji W.J., Zhou Q. (2021). LncRNA HCP5 in hBMSC-derived exosomes alleviates myocardial ischemia reperfusion injury by sponging miR-497 to activate IGF1/PI3K/AKT pathway. Int. J. Cardiol..

[41] Sun X.H., Wang X., Zhang Y., Hui J. (2019). Exosomes of bone-marrow stromal cells inhibit cardiomyocyte apoptosis under ischemic and hypoxic conditions via miR-486-5p targeting the PTEN/PI3K/AKT signaling pathway. Thromb. Res..

[42] Zhang J., Zhang X. (2021). Ischaemic preconditioning-induced serum exosomes protect against myocardial ischaemia/reperfusion injury in rats by activating the PI3K/AKT signalling pathway. Cell Biochem. Funct..

[43] Zheng L., Li Z., Ling W., Zhu D., Feng Z., Kong L. (2018). Exosomes Derived from Dendritic Cells Attenuate Liver Injury by Modulating the Balance of Treg and Th17 Cells After Ischemia Reperfusion. Cell Physiol. Biochem..

[44] Liu H., Shi M., Li X., Lu W., Zhang M., Zhang T., Wu Y., Zhang Z., Cui Q., Yang S. (2022). Adipose Mesenchymal Stromal Cell-Derived Exosomes Prevent Testicular Torsion Injury via Activating PI3K/AKT and MAPK/ERK1/2 Pathways. Oxid. Med. Cell. Longev..

[45] Zhang C., Wang H., Chan G.C.F., Zhou Y., Lai X., Lian M. (2020). Extracellular Vesicles Derived from Human Umbilical Cord Mesenchymal Stromal Cells Protect Cardiac Cells Against Hypoxia/Reoxygenation Injury by Inhibiting Endoplasmic Reticulum Stress via Activation of the PI3K/Akt Pathway. Cell Transpl..

[46] Dai Y., Wang S., Chang S., Ren D., Shali S., Li C., Yang H., Huang Z., Ge J. (2020). M2 macrophage-derived exosomes carry microRNA-148a to alleviate myocardial ischemia/reperfusion injury via inhibiting TXNIP and the TLR4/NF-κB/NLRP3 inflammasome signaling pathway. J. Mol. Cell. Cardiol..

[47] Wang K., Ru J., Zhang H., Chen J., Lin X., Lin Z., Wen M., Huang L., Ni H., Zhuge Q. (2020). Melatonin Enhances the Therapeutic Effect of Plasma Exosomes Against Cerebral Ischemia-Induced Pyroptosis Through the TLR4/NF-κB Pathway. Front. Neurosci..

[48] Wei W., Li H., Deng Y., Zheng X., Zhou Y., Xue X. (2023). The combination of Alisma and Atractylodes ameliorates cerebral ischaemia/reperfusion injury by negatively regulating astrocyte-derived exosomal miR-200a-3p/141-3p by targeting SIRT1. J. Ethnopharmacol..

[49] Senda A., Morishita K., Kojima M., Doki S., Taylor B., Yagi M., Watanabe A., Kobayashi T., Aiboshi J., Coimbra R. (2020). The role of mesenteric lymph exosomal lipid mediators following intestinal ischemia-reperfusion injury on activation of inflammation. J. Trauma. Acute Care Surg..

[50] Li X., Liao J., Su X., Li W., Bi Z., Wang J., Su Q., Huang H., Wei Y., Gao Y. (2020). Human urine-derived stem cells protect against renal ischemia/reperfusion injury in a rat model via exosomal miR-146a-5p which targets IRAK1. Theranostics.

[51] Pan T., Jia P., Chen N., Fang Y., Liang Y., Guo M., Ding X. (2019). Delayed Remote Ischemic Preconditioning ConfersRenoprotection against Septic Acute Kidney Injury via Exosomal miR-21. Theranostics.

[52] Guo L., Huang Z., Huang L., Liang J., Wang P., Zhao L., Shi Y. (2021). Surface-modified engineered exosomes attenuated cerebral ischemia/reperfusion injury by targeting the delivery of quercetin towards impaired neurons. J. Nanobiotechnol..

[53] Liu Q., Tan Y., Qu T., Zhang J., Duan X., Xu H., Mu Y., Ma H., Wang F. (2020). Therapeutic mechanism of human neural stem cell-derived extracellular vesicles against hypoxia-reperfusion injury in vitro. Life Sci..

[54] Xiao T., Qu H., Zeng Z., Li C., Wan J. (2022). Exosomes from M2-polarized macrophages relieve oxygen/glucose deprivation/normalization-induced neuronal injury by activating the Nrf2/HO-1 signaling. Arch. Biochem. Biophys..

[55] Cao H., Cheng Y., Gao H., Zhuang J., Zhang W., Bian Q., Wang F., Du Y., Li Z., Kong D. (2020). In Vivo Tracking of Mesenchymal Stem Cell-Derived Extracellular Vesicles Improving Mitochondrial Function in Renal Ischemia-Reperfusion Injury. ACS Nano.

[56] Zhang J., Su R., Wang Y., Wang H., Li S., Yang X., Liu G. (2024). Protective effect of small extracellular vesicles (EVs) derived from ACE2-modified human umbilical cord mesenchymal stem cells against renal ischemia-reperfusion injury. Nephrology.

[57] Li T., Gu J., Yang O., Wang J., Wang Y., Kong J. (2020). Bone Marrow Mesenchymal Stem Cell-Derived Exosomal miRNA-29c Decreases Cardiac Ischemia/Reperfusion Injury Through Inhibition of Excessive Autophagy via the PTEN/Akt/mTOR Signaling Pathway. Circ. J..

[58] Wei Z., Chen Z., Zhao Y., Fan F., Xiong W., Song S., Yin Y., Hu J., Yang K., Yang L. (2021). Mononuclear phagocyte system blockade using extracellular vesicles modified with CD47 on membrane surface for myocardial infarction reperfusion injury treatment. Biomaterials.

[59] Zhang Y., Wang J., Yang B., Qiao R., Li A., Guo H., Ding J., Li H., Ye H., Wu D. (2020). Transfer of MicroRNA-216a-5p From Exosomes Secreted by Human Urine-Derived Stem Cells Reduces Renal Ischemia/Reperfusion Injury. Front. Cell Dev. Biol..

[60] Li J.W., Wei L., Han Z., Chen Z. (2019). Mesenchymal stromal cells-derived exosomes alleviate ischemia/reperfusion injury in mouse lung by transporting anti-apoptotic miR-21-5p. Eur. J. Pharmacol..

[61] Zhang G., Wan Z., Liu Z., Liu D., Zhao Z., Leng Y. (2022). Exosomes Derived from BMSCs Ameliorate Intestinal Ischemia-Reperfusion Injury by Regulating miR-144-3p-Mediated Oxidative Stress. Dig. Dis. Sci..

[62] Li H., Lin W., Zhang G., Liu R., Qu M., Zhang J., Xing X. (2023). BMSC-exosomes miR-25-3p Regulates the p53 Signaling Pathway Through PTEN to Inhibit Cell Apoptosis and Ameliorate Liver Ischemia-reperfusion Injury. Stem Cell Rev. Rep..

[63] Li X., Zhang Y., Wang Y., Zhao D., Sun C., Zhou S., Xu D., Zhao J. (2020). Exosomes Derived from CXCR4-Overexpressing BMSC Promoted Activation of Microvascular Endothelial Cells in Cerebral Ischemia/Reperfusion Injury. Neural Plast..

[64] Cui X., He Z., Liang Z., Chen Z., Wang H., Zhang J. (2017). Exosomes From Adipose-derived Mesenchymal Stem Cells Protect the Myocardium Against Ischemia/Reperfusion Injury Through Wnt/β-Catenin Signaling Pathway. J. Cardiovasc. Pharmacol..

[65] Zou L., Ma X., Wu B., Chen Y., Xie D., Peng C. (2020). Protective effect of bone marrow mesenchymal stem cell-derived exosomes on cardiomyoblast hypoxia-reperfusion injury through the miR-149/let-7c/Faslg axis. Free Radic. Res..

[66] Park H., Park H., Mun D., Kang J., Kim H., Kim M., Cui S., Lee S.H., Joung B. (2018). Extracellular Vesicles Derived from Hypoxic Human Mesenchymal Stem Cells Attenuate GSK3β Expression via miRNA-26a in an Ischemia-Reperfusion Injury Model. Yonsei Med. J..

[67] Tian T., Cao L., He C., Ye Q., Liang R., You W., Zhang H., Wu J., Ye J., Tannous B.A. (2021). Targeted delivery of neural progenitor cell-derived extracellular vesicles for anti-inflammation after cerebral ischemia. Theranostics.

[68] Wu W., Liu J., Yang C., Xu Z., Huang J., Lin J. (2020). Astrocyte-derived exosome-transported microRNA-34c is neuroprotective against cerebral ischemia/reperfusion injury via TLR7 and the NF-κB/MAPK pathways. Brain Res. Bull..

[69] Zhang Y., Li Y., Wang Q., Zheng D., Feng X., Zhao W., Cai L., Zhang Q., Xu H., Fu H. (2022). Attenuation of hepatic ischemia-reperfusion injury by adipose stem cell-derived exosome treatment via ERK1/2 and GSK-3β signaling pathways. Int. J. Mol. Med..

[70] Gong Y., Dai H., Liu W., Liao R., Chen H., Zhang L., Wang X., Chen Z. (2023). Exosomes derived from human adipose-derived stem cells alleviate hepatic ischemia-reperfusion (I/R) injury through the miR-183/ALOX5 axis. FASEB J. Off. Publ. Fed. Am. Soc. Exp. Biol..

[71] Kang X., Chen Y., Xin X., Liu M., Ma Y., Ren Y., Ji J., Yu Q., Qu L., Wang S. (2021). Human Amniotic Epithelial Cells and Their Derived Exosomes Protect Against Cisplatin-Induced Acute Kidney Injury Without Compromising Its Antitumor Activity in Mice. Front. Cell Dev. Biol..

[72] Zhao H., Chen X., Hu G., Li C., Guo L., Zhang L., Sun F., Xia Y., Yan W., Cui Z. (2022). Small Extracellular Vesicles From Brown Adipose Tissue Mediate Exercise Cardioprotection. Circ. Res..

[73] Jiang Y., He R., Shi Y., Liang J., Zhao L. (2020). Plasma exosomes protect against cerebral ischemia/reperfusion injury via exosomal HSP70 mediated suppression of ROS. Life Sci..

[74] Li G., Xiao L., Qin H., Zhuang Q., Zhang W., Liu L., Di C., Zhang Y. (2020). Exosomes-carried microRNA-26b-5p regulates microglia M1 polarization after cerebral ischemia/reperfusion. Cell Cycle.

[75] Li X., Bi T., Yang S. (2022). Exosomal microRNA-150-5p from bone marrow mesenchymal stromal cells mitigates cerebral ischemia/reperfusion injury via targeting toll-like receptor 5. Bioengineered.

[76] Zhang L., Wei Q., Liu X., Zhang T., Wang S., Zhou L., Zou L., Fan F., Chi H., Sun J. (2021). Exosomal microRNA-98-5p from hypoxic bone marrow mesenchymal stem cells inhibits myocardial ischemia-reperfusion injury by reducing TLR4 and activating the PI3K/Akt signaling pathway. Int. Immunopharmacol..

[77] Zheng S., Wang L., Ma H., Sun F., Wen F. (2021). microRNA-129 overexpression in endothelial cell-derived extracellular vesicle influences inflammatory response caused by myocardial ischemia/reperfusion injury. Cell Biol. Int..

[78] Zeng Q., Zhou Y., Liang D., He H., Liu X., Zhu R., Zhang M., Luo X., Wang Y., Huang G. (2020). Exosomes Secreted From Bone Marrow Mesenchymal Stem Cells Attenuate Oxygen-Glucose Deprivation/Reoxygenation-Induced Pyroptosis in PC12 Cells by Promoting AMPK-Dependent Autophagic Flux. Front. Cell Neurosci..

[79] Liu L., Jin X., Hu C.F., Li R., Zhou Z., Shen C.X. (2017). Exosomes Derived from Mesenchymal Stem Cells Rescue Myocardial Ischaemia/Reperfusion Injury by Inducing Cardiomyocyte Autophagy Via AMPK and Akt Pathways. Cell. Physiol. Biochem..

[80] Su Q., Lv X.W., Xu Y.L., Cai R.P., Dai R.X., Yang X.H., Zhao W.K., Kong B.H. (2021). Exosomal LINC00174 derived from vascular endothelial cells attenuates myocardial I/R injury via p53-mediated autophagy and apoptosis. Mol. Ther. Nucleic Acids.

[81] Han M., Cao Y., Xue H., Chu X., Li T., Xin D., Yuan L., Ke H., Li G., Wang Z. (2020). Neuroprotective Effect of Mesenchymal Stromal Cell-Derived Extracellular Vesicles Against Cerebral Ischemia-Reperfusion-Induced Neural Functional Injury: A Pivotal Role for AMPK and JAK2/STAT3/NF-κB Signaling Pathway Modulation. Drug Des. Devel Ther..

[82] Tao H., Li L., Dong L., Chen H., Shan X., Zhuge L., Lou H. (2023). Growth differentiation factor 7 pretreatment enhances the therapeutic capacity of bone marrow-derived mesenchymal stromal cells against cerebral ischemia-reperfusion injury. Chem.-Biol. Interact..

[83] Xu F., Na L., Li Y., Chen L. (2020). Roles of the PI3K/AKT/mTOR signalling pathways in neurodegenerative diseases and tumours. Cell Biosci..

[84] Glaviano A., Foo A.S.C., Lam H.Y., Yap K.C.H., Jacot W., Jones R.H., Eng H., Nair M.G., Makvandi P., Geoerger B. (2023). PI3K/AKT/mTOR signaling transduction pathway and targeted therapies in cancer. Mol. Cancer.

[85] Deng R.M., Zhou J. (2023). The role of PI3K/AKT signaling pathway in myocardial ischemia-reperfusion injury. Int. Immunopharmacol..

[86] Ruan C., Guo H., Gao J., Wang Y., Liu Z., Yan J., Li X., Lv H. (2021). Neuroprotective effects of metformin on cerebral ischemia-reperfusion injury by regulating PI3K/Akt pathway. Brain Behav..

[87] Feng C., Wan H., Zhang Y., Yu L., Shao C., He Y., Wan H., Jin W. (2020). Neuroprotective Effect of Danhong Injection on Cerebral Ischemia-Reperfusion Injury in Rats by Activation of the PI3K-Akt Pathway. Front. Pharmacol..

[88] Wang M., Zhang J., Zhang J., Sun K., Li Q., Kuang B., Wang M.M.Z., Hou S., Gong N. (2021). Methyl eugenol attenuates liver ischemia reperfusion injury via activating PI3K/Akt signaling. Int. Immunopharmacol..

[89] Oh A., Pardo M., Rodriguez A., Yu C., Nguyen L., Liang O., Chorzalska A., Dubielecka P.M. (2023). NF-κB signaling in neoplastic transition from epithelial to mesenchymal phenotype. Cell Commun. Signal. CCS.

[90] Dong P., Liu K., Han H. (2022). The Role of NF-κB in Myocardial Ischemia/Reperfusion Injury. Curr. Protein Pept. Sci..

[91] Dong X., Wang L., Song G., Cai X., Wang W., Chen J., Wang G. (2021). Physcion Protects Rats Against Cerebral Ischemia-Reperfusion Injury via Inhibition of TLR4/NF-kB Signaling Pathway. Drug Des. Dev. Ther..

[92] Li M., Hou Q., Zhong L., Zhao Y., Fu X. (2021). Macrophage Related Chronic Inflammation in Non-Healing Wounds. Front. Immunol..

[93] Tsai S.C., Yang K.D., Chang K.H., Lin F.C., Chou R.H., Li M.C., Cheng C.C., Kao C.Y., Chen C.P., Lin H.C. (2021). Umbilical Cord Mesenchymal Stromal Cell-Derived Exosomes Rescue the Loss of Outer Hair Cells and Repair Cochlear Damage in Cisplatin-Injected Mice. Int. J. Mol. Sci..

[94] Cao J.Y., Wang B., Tang T.T., Wen Y., Li Z.L., Feng S.T., Wu M., Liu D., Yin D., Ma K.L. (2021). Exosomal miR-125b-5p deriving from mesenchymal stem cells promotes tubular repair by suppression of p53 in ischemic acute kidney injury. Theranostics.

[95] Kim S., Lee S.A., Yoon H., Kim M.Y., Yoo J.K., Ahn S.H., Park C.H., Park J., Nam B.Y., Park J.T. (2021). Exosome-based delivery of super-repressor IκBα ameliorates kidney ischemia-reperfusion injury. Kidney Int..

[96] Lin L., Wu Q., Lu F., Lei J., Zhou Y., Liu Y., Zhu N., Yu Y., Ning Z., She T. (2023). Nrf2 signaling pathway: Current status and potential therapeutic targetable role in human cancers. Front. Oncol..

[97] Li B., Wang Y., Jiang X., Du H., Shi Y., Xiu M., Liu Y., He J. (2023). Natural products targeting Nrf2/ARE signaling pathway in the treatment of inflammatory bowel disease. Biomed. Pharmacother..

[98] Ucar B.I., Ucar G., Saha S., Buttari B., Profumo E., Saso L. (2021). Pharmacological Protection against Ischemia-Reperfusion Injury by Regulating the Nrf2-Keap1-ARE Signaling Pathway. Antioxidants.

[99] Ya B.L., Liu Q., Li H.F., Cheng H.J., Yu T., Chen L., Wang Y., Yuan L.L., Li W.J., Liu W.Y. (2018). Uric Acid Protects against Focal Cerebral Ischemia/Reperfusion-Induced Oxidative Stress via Activating Nrf2 and Regulating Neurotrophic Factor Expression. Oxidative Med. Cell. Longev..

[100] Raquel G.B., Panisello-Roselló A., Sanchez-Nuno S., Alva N., Roselló-Catafau J., Carbonell T. (2022). Nrf2 and oxidative stress in liver ischemia/reperfusion injury. FEBS J..

[101] Liu W., Tang P., Wang J., Ye W., Ge X., Rong Y., Ji C., Wang Z., Bai J., Fan J. (2021). Extracellular vesicles derived from melatonin-preconditioned mesenchymal stem cells containing USP29 repair traumatic spinal cord injury by stabilizing NRF2. J. Pineal Res..

[102] Cui X., Liu X., Kong P., Du T., Li T., Yang G., Zhang W., Jing X., Wang W. (2023). PTEN inhibitor VO-OHpic protects endplate chondrocytes against apoptosis and calcification via activating Nrf-2 signaling pathway. Aging.

[103] Jiang T.Y., Shi Y.Y., Cui X.W., Pan Y.F., Lin Y.K., Feng X.F., Ding Z.W., Yang C., Tan Y.X., Dong L.W. (2023). PTEN Deficiency Facilitates Exosome Secretion and Metastasis in Cholangiocarcinoma by Impairing TFEB-mediated Lysosome Biogenesis. Gastroenterology.

[104] Xu Y., Tang Y., Lu J., Zhang W., Zhu Y., Zhang S., Ma G., Jiang P., Zhang W. (2020). PINK1-mediated mitophagy protects against hepatic ischemia/reperfusion injury by restraining NLRP3 inflammasome activation. Free Radic. Biol. Med..

[105] Du J., Li Y., Zhao W. (2020). Autophagy and Myocardial Ischemia. Adv. Exp. Med. Biol..

[106] Xu X., Zhang M., Xu F., Jiang S. (2020). Wnt signaling in breast cancer: Biological mechanisms, challenges and opportunities. Mol. Cancer.

[107] Ma Q., Yu J., Zhang X., Wu X., Deng G. (2023). Wnt/β-catenin signaling pathway-a versatile player in apoptosis and autophagy. Biochimie.

[108] Liu J., Xiao Q., Xiao J., Niu C., Li Y., Zhang X., Zhou Z., Shu G., Yin G. (2022). Wnt/β-catenin signalling: Function, biological mechanisms, and therapeutic opportunities. Signal Transduct. Target. Ther..

[109] Ji Y.B., Gao Q., Tan X.X., Huang X.W., Ma Y.Z., Fang C., Wang S.N., Qiu L.H., Cheng Y.X., Guo F.Y. (2021). Lithium alleviates blood-brain barrier breakdown after cerebral ischemia and reperfusion by upregulating endothelial Wnt/β-catenin signaling in mice. Neuropharmacology.

[110] Zhao H., Ming T., Tang S., Ren S., Yang H., Liu M., Tao Q., Xu H. (2022). Wnt signaling in colorectal cancer: Pathogenic role and therapeutic target. Mol. Cancer.

[111] Wang Y., Cao Z., Liu F., Ou Y. (2021). Clinical significance of activated Wnt/β-catenin signaling in apoptosis inhibition of oral cancer. Open Life Sci..

[112] Piao C., Sang J., Kou Z., Wang Y., Liu T., Lu X., Jiao Z., Wang H. (2022). Effects of Exosomes Derived from Adipose-Derived Mesenchymal Stem Cells on Pyroptosis and Regeneration of Injured Liver. Int. J. Mol. Sci..

[113] Ma Y., Nicolet J. (2023). Specificity models in MAPK cascade signaling. FEBS Open Bio.

[114] Qi X.M., Chen G. (2023). p38γ MAPK Inflammatory and Metabolic Signaling in Physiology and Disease. Cells.

[115] Xu D., Kong T., Shao Z., Liu M., Zhang R., Zhang S., Kong Q., Chen J., Cheng B., Wang C. (2021). Orexin-A alleviates astrocytic apoptosis and inflammation via inhibiting OX1R-mediated NF-κB and MAPK signaling pathways in cerebral ischemia/reperfusion injury. Biochim. Biophys. Acta Mol. Basis Dis..

[116] Huang L., Li X., Liu Y., Liang X., Ye H., Yang C., Hua L., Zhang X. (2021). Curcumin Alleviates Cerebral Ischemia-reperfusion Injury by Inhibiting NLRP1-dependent Neuronal Pyroptosis. Curr. Neurovasc Res..

[117] Zhou Q.L., Teng F., Zhang Y.S., Sun Q., Cao Y.X., Meng G.W. (2018). FPR1 gene silencing suppresses cardiomyocyte apoptosis and ventricular remodeling in rats with ischemia/reperfusion injury through the inhibition of MAPK signaling pathway. Exp. Cell Res..

[118] Zhang Y., Wu J., Dong E., Wang Z., Xiao H. (2023). Toll-like receptors in cardiac hypertrophy. Front. Cardiovasc. Med..

[119] Zhang Y., Liu J., Wang C., Liu J., Lu W. (2021). Toll-Like Receptors Gene Polymorphisms in Autoimmune Disease. Front. Immunol..

[120] Chu Y.T., Liao M.T., Tsai K.W., Lu K.C., Hu W.C. (2023). Interplay of Chemokines Receptors, Toll-like Receptors, and Host Immunological Pathways. Biomedicines.

[121] Hu J., Deng F., Zhao B., Lin Z., Sun Q., Yang X., Wu M., Qiu S., Chen Y., Yan Z. (2022). Lactobacillus murinus alleviate intestinal ischemia/reperfusion injury through promoting the release of interleukin-10 from M2 macrophages via Toll-like receptor 2 signaling. Microbiome.

[122] Kitazume-Taneike R., Taneike M., Omiya S., Misaka T., Nishida K., Yamaguchi O., Akira S., Shattock M.J., Sakata Y., Otsu K. (2019). Ablation of Toll-like receptor 9 attenuates myocardial ischemia/reperfusion injury in mice. Biochem. Biophys. Res. Commun..

[123] Guo L.L., Guo M.L., Yao J., Weng Y.Q., Zhang X.Z. (2020). MicroRNA-421 improves ischemia/reperfusion injury via regulation toll-like receptor 4 pathway. J. Int. Med. Res..

[124] Mentor S., Fisher D. (2022). Exosomes form tunneling nanotubes (TUNTs) in the blood-brain barrier: A nano-anatomical perspective of barrier genesis. Front. Mol. Neurosci..

[125] Gao X., Lin C., Feng Y., You Y., Jin Z., Li M., Zhou Y., Chen K. (2023). Akkermansia muciniphila-derived small extracellular vesicles attenuate intestinal ischemia-reperfusion-induced postoperative cognitive dysfunction by suppressing microglia activation via the TLR2/4 signaling. Biochim. Biophys. Acta. Mol. Cell Res..

[126] Zhang T., Xu D., Trefts E., Lv M., Inuzuka H., Song G., Liu M., Lu J., Liu J., Chu C. (2023). Metabolic orchestration of cell death by AMPK-mediated phosphorylation of RIPK1. Science.

[127] Lu Y., Zhang C., Chen J., Zou Q., Li B., Wei H., Chang M.P., Liao X., Hu C. (2022). Hypothermia preconditioning improves cardiac contractility after cardiopulmonary resuscitation through AMPK-activated mitophagy. Exp. Biol. Med..

[128] Zuurbier C.J., Bertrand L., Beauloye C.R., Andreadou I., Ruiz-Meana M., Jespersen N.R., Kula-Alwar D., Prag H.A., Eric Botker H., Dambrova M. (2020). Cardiac metabolism as a driver and therapeutic target of myocardial infarction. J. Cell. Mol. Med..

[129] Liu H., Wu X., Luo J., Zhao L., Li X., Guo H., Bai H., Cui W., Guo W., Feng D. (2020). Adiponectin peptide alleviates oxidative stress and NLRP3 inflammasome activation after cerebral ischemia-reperfusion injury by regulating AMPK/GSK-3β. Exp. Neurol..

[130] Li D., Zhao Y., Zhang C., Wang F., Zhou Y., Jin S. (2021). Plasma Exosomes at the Late Phase of Remote Ischemic Pre-conditioning Attenuate Myocardial Ischemia-Reperfusion Injury Through Transferring miR-126a-3p. Front. Cardiovasc. Med..

[131] Wang Y., Shen Y. (2022). Exosomal miR-455-3p from BMMSCs prevents cardiac ischemia-reperfusion injury. Hum. Exp. Toxicol..

[132] Yang D., Wang M., Hu Z., Ma Y., Shi Y., Cao X., Guo T., Cai H., Cai H. (2021). Extracorporeal Cardiac Shock Wave-Induced Exosome Derived From Endothelial Colony-Forming Cells Carrying miR-140-3p Alleviate Cardiomyocyte Hypoxia/Reoxygenation Injury via the PTEN/PI3K/AKT Pathway. Front. Cell Dev. Biol..

[133] Douvris A., Viñas J., Burns K.D. (2022). miRNA-486-5p: Signaling targets and role in non-malignant disease. Cell. Mol. Life Sci..

[134] Zhang L., Liu H., Xu K., Ling Z., Huang Y., Hu Q., Lu K., Liu C., Wang Y., Liu N. (2019). Hypoxia preconditioned renal tubular epithelial cell-derived extracellular vesicles alleviate renal ischaemia-reperfusion injury mediated by the HIF-1α/Rab22 pathway and potentially affected by microRNAs. Int. J. Biol. Sci..

[135] Zheng J., Lu T., Zhou C., Cai J., Zhang X., Liang J., Sui X., Chen X., Chen L., Sun Y. (2020). Extracellular Vesicles Derived from Human Umbilical Cord Mesenchymal Stem Cells Protect Liver Ischemia/Reperfusion Injury by Reducing CD154 Expression on CD4+ T Cells via CCT2. Adv. Sci..

[136] Zeng X., Zhang Y.D., Ma R.Y., Chen Y.J., Xiang X.M., Hou D.Y., Li X.H., Huang H., Li T., Duan C.Y. (2022). Activated Drp1 regulates p62-mediated autophagic flux and aggravates inflammation in cerebral ischemia-reperfusion via the ROS-RIP1/RIP3-exosome axis. Mil. Med. Res..

[137] Fan M., Sun W., Gu X., Lu S., Shen Q., Liu X., Zhang X. (2022). The critical role of STAT3 in biogenesis of tumor-derived exosomes with potency of inducing cancer cachexia in vitro and in vivo. Oncogene.

[138] Wei M., Gao X., Liu L., Li Z., Wan Z., Dong Y., Chen X., Niu Y., Zhang J., Yang G. (2020). Visceral Adipose Tissue Derived Exosomes Exacerbate Colitis Severity via Pro-inflammatory MiRNAs in High Fat Diet Fed Mice. ACS Nano.

[139] Dehkordi N.R., Dehkordi N.R., Farjoo M.H. (2022). Therapeutic properties of stem cell-derived exosomes in ischemic heart disease. Eur. J. Pharmacol..

[140] Xin W., Qin Y., Lei P., Zhang J., Yang X., Wang Z. (2022). From cerebral ischemia towards myocardial, renal, and hepatic ischemia: Exosomal miRNAs as a general concept of intercellular communication in ischemia-reperfusion injury. Mol. Ther. Nucleic Acids.

[141] Zheng X.F., Zhang X.J., Dong L.P., Zhao J.R., Zhang C., Chen R. (2023). Neuroprotective mechanism of salvianolic acid B against cerebral ischemia-reperfusion injury in mice through downregulation of TLR4, p-p38MAPK, p-JNK, NF-κB, and IL-1β. Immun. Inflamm. Dis..

